# Structure and Function of the Dental Plaque Microbiome in Eubiosis: A Systematic Review of Ethnic-Racial Influences

**DOI:** 10.3390/microorganisms14051095

**Published:** 2026-05-12

**Authors:** Edisson Ronaldo Duran Yunga, María de Lourdes Rodriguez Coyago

**Affiliations:** 1 Facultad de Odontología, Universidad de Cuenca, Cuenca 010107, Ecuador; edisson.duran@ucuenca.edu.ec; 2 Departamento de Microbiología y Diagnóstico, Facultad de Odontología, Universidad de Cuenca, Cuenca 010107, Ecuador; 3 Grupo de Investigación en Rehabilitación Oral (GIRO), Facultad de Odontología, Universidad de Cuenca, Cuenca 010107, Ecuador

**Keywords:** supragingival plaque, oral microbiome, eubiosis, ethnicity, systematic review, personalized dentistry

## Abstract

While a conserved core microbiome is shared across healthy individuals, significant interindividual taxonomic variation exists; however, the specific influence of genetic ancestry on supragingival plaque structure in eubiosis remains unclear. This systematic review analyzed evidence regarding taxonomic variations in supragingival plaque associated with ethnicity in systemically healthy populations. A search was conducted in PubMed, Scopus, ScienceDirect, and Scielo following PRISMA 2020 guidelines, covering literature up to October 2025. Cross-sectional studies using genomic sequencing or metagenomics were included, with quality assessed via the GRADE system. Six studies met eligibility criteria. Results identified a universal core microbiome structurally dominated by *Corynebacterium* spp. and *Streptococcus* spp. However, distinct ethnic-specific taxonomic signatures emerged, such as the enrichment of *Fusobacterium* spp. in African Americans and *Corynebacterium* spp. in Caucasians, alongside the exclusive presence of *Sneathia* spp. in Burmese individuals. Although a basal microbial architecture necessary for homeostasis exists, ethnicity acts as a biological filter defining distinctive bacterial profiles and differential susceptibilities. These findings suggest that while the core microbiome is conserved, the composition of peripheral species in the dental plaque hedgehog structure varies according to ancestry. This supports a transition from standardized dental care to personalized medicine oriented towards the patient’s biological heritage.

## 1. Introduction

The human oral cavity harbors one of the most diverse microbial communities in the body, with an estimated 700 bacterial species or phylotypes [1]. These communities do not exist as plankton, but rather as highly organized biofilms attached to surfaces and embedded in an extracellular matrix [2,3,4]. In a state of health or eubiosis, these microbial assemblages contribute to host homeostasis [5], modulating immune responses and participating in crucial metabolic functions, as long as this community remains diverse and balanced, predominated by taxa considered symbiotic or commensal [6].

Supragingival dental plaque is a dynamic structured biofilm that adheres to the tooth surface [2]. This microbial ecosystem is one of the most densely populated and metabolically active sites in the human body [1], acting as the main interface between the external environment (diet and hygiene) and the internal environment (genetics and immune response) [5]. Understanding its structure and function is fundamental, as its alteration or dysbiosis is directly correlated with the two most prevalent oral pathologies worldwide: dental caries and periodontal disease [7].

There is a clinical consensus that diseases such as periodontitis disproportionately affect certain racial/ethnic populations [8]. While social determinants of health (access to care, dietary and behavioral factors) explain much of these disparities, a growing body of research postulates that innate variations in oral microbial communities also play a fundamental role [9]. The central hypothesis is that, even in clinically healthy individuals, the composition and functional potential of the basal microbiome may differ significantly between ethnic groups [10], implying that some groups may start with an eubiosis configuration that is inherently more vulnerable [9] or “primed” for dysbiotic change in response to environmental or systemic stressors [11].

Understanding the composition and function of a healthy microbiome in different ethnicities is essential to establish baseline measures of eubiosis [5]. The assumption that the composition of healthy biofilms is similar across all populations has guided treatment protocols for years [6]; however, if communities compatible with health consist of different types or proportions of species depending on ethnicity, prevention and treatment strategies need to be reevaluated [12].

Against this backdrop, a systematic review of cross-sectional observational studies is proposed, with the aim of establishing a consensus on the existence of variations in the structure and function of the healthy dental plaque microbiome attributable to ethnicity or ancestry.

## 2. Materials and Methods

### 2.1. Search Strategy and Data Sources

The PRISMA (Preferred Reporting Items for Systematic Reviews and Meta-Analyses) 2020 guideline [13] was used to systematize the search and selection of evidence to answer the formulated research question: Are there consistent variations in the taxonomic structure and functional potential of the healthy dental plaque microbiome attributable to ethnicity or ancestry? To address this research question, the definitions of ethnicity and race provided by Borrell et al. [14] and Oni-Orisan et al. [15] were adopted as theoretical frameworks. Four globally recognized biomedical databases were consulted: PubMed, ScienceDirect, Scopus, and SciELO. To minimize potential language bias during the identification phase, no language restrictions or filters were applied within the database search engines. The search period covered publications from January 2005 to October 2025. The protocol for this systematic review was pre-registered on the Open Science Framework (OSF) platform (Registration DOI: https://doi.org/10.17605/OSF.IO/5Y4W7 accessed on 29 March 2026). Search terms (MeSH and free text) were combined using Boolean operators, focusing on the intersection of oral microbiology, health, and demography. To ensure full methodological transparency, the exact search strings applied to each database, along with the traceability of the screening process, are detailed in Supplementary Table S1.

### 2.2. Eligibility Criteria

Strict eligibility criteria were applied based on the PICOS framework:

- Population (P): Human subjects older than 2 years (an age threshold selected because the oral microbiome stabilizes and reaches an adult-like composition following the establishment of primary dentition [16]) who were clinically healthy or free from the specific medical or oral pathology of interest.
- Exposure (I/E): Subjects exposed to a particular racial influence: African Americans (AAs), Caucasians (CAs), Hispanic Americans (HAs), Asians (ASs), Burmese (BM), and Australians (AUSs).
- Comparison (C): Cross-sectional observational studies that made a direct comparison between two or more ethnic, racial, or distinct geographic populations, and studies comparing oral health conditions within a single racial group through supragingival dental plaque sampling. Where reported by the primary authors, potential confounding variables (e.g., diet, environmental factors) were extracted to contextualize these comparisons.
- Outcome (O): Characterization of the structure (alpha and beta diversity, taxonomic composition, abundance) and/or function (functional prediction, metagenomics) of the supragingival dental plaque microbiome.

Primary articles were included if they met the following criteria:

- Cross-sectional observational design;
- Analysis of supragingival dental plaque using high-throughput sequencing (16S rRNA or Shotgun Metagenomics);
- Inclusion of at least one defined ethnic/racial group (e.g., AA, CA, AS, HA);
- The main study cohort had to be classified as systemically and orally healthy (no dental caries, periodontal disease, or structural defects of the dental enamel).

The following study types and conditions were excluded:

- Literature reviews;
- Studies correlating plaque structure and composition in patients with systemic diseases;
- Analytical cohort and case–control studies;
- Clinical trials;
- Animal model studies;
- Letters to the editor;
- In vitro studies.

**Figure 1:**
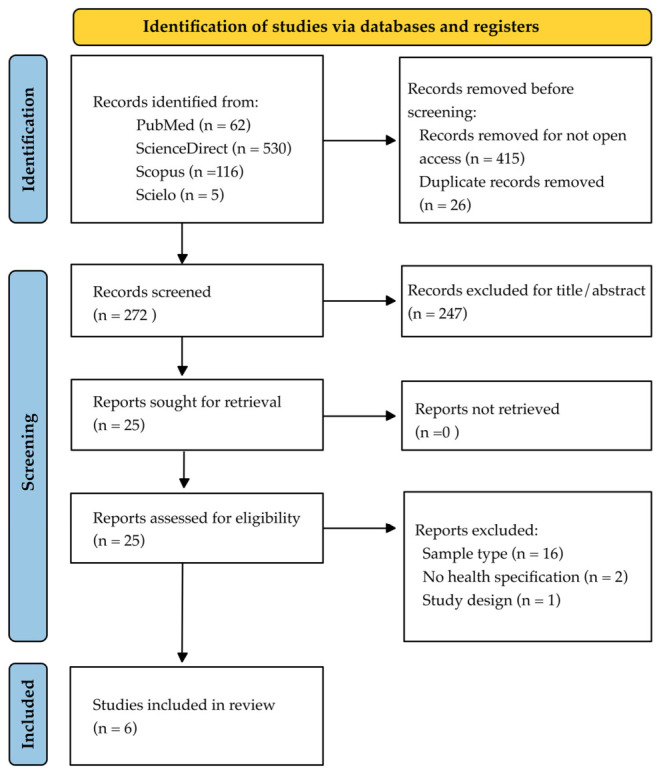
PRISMA 2020 flow diagram for the systematic review selection process. The diagram illustrates the number of records identified, included, and excluded, and the reasons for exclusions at each stage, adhering to the PRISMA 2020 guidelines.

Cross-sectional observational studies were exclusively selected because they provide an accurate point-in-time ‘snapshot’ of the oral microbiome in a confirmed state of eubiosis. Longitudinal or cohort studies were excluded as their primary focus is typically tracking disease progression, incidence, or treatment outcomes over time, which inherently introduces temporal variables and shifts in clinical health status that fall outside the scope of defining a strictly healthy baseline. Figure 1 schematically summarizes the process of searching for and selecting evidence.

### 2.3. Definition of Oral Health and Ethnic-Racial Cohorts

To ensure that the microbial differences found could be attributed to ethnicity and not to subclinical inflammation or disease, priority was given to studies that applied rigorous clinical and exclusion criteria. The gold standard for periodontal health was defined as: absence of systemic diseases (e.g., diabetes, HIV), exclusion of smokers, no recent use of antibiotics or steroids (within 3 months), and clinical criteria such as probing pocket depth (PPD) equal to or less than 3 mm at all sites [17], and a Plaque Index (PI) according to the Löe plaque index equal to or less than 1.5 [18].

Ethnic cohorts were defined based on self-identification or confirmation that both parents and grandparents belonged to the same ethnic group.

### 2.4. Data Extraction and Quality Assessment

Data extraction focused on key quantitative metrics of microbial ecology. This included alpha diversity indices (e.g., Shannon, Chao1), beta diversity outcomes (e.g., PCoA, PERMANOVA based on UniFrac distances), the relative abundance of the most dominant phyla and genera, and findings from functional predictions (e.g., PICRUSt or HUMAnN comparing KEGG pathways). Selected studies were evaluated for their level of evidence under the GRADE guidelines [19].

### 2.5. Study Selection

The selection process was carried out independently and in duplicate. Two reviewers (ERDY and MLRC) evaluated the titles and abstracts of each study retrieved from the aforementioned databases. Only one reviewer (ERDY) performed the full-text reading of each article selected according to the inclusion criteria. Only in cases of unclear data did the second reviewer (MLRC) participate.

### 2.6. Data Acquisition

Process sociodemographic data and variables relevant to answering the PICO question were collected and organized in a table using Microsoft Excel 2019 (Microsoft Corp., Redmond, WA, USA). The data extraction process was carried out independently by two reviewers, involving a thorough reading of methodology and results. Studies that were excluded after full-text review were recorded in a database indicating the reasons for exclusion (Supplementary Table S2).

### 2.7. Data of Interest

The following variables were obtained from each selected article: number of participants, age range, ethnic groups studied, identification method, percentage of shared OTUs (operational taxonomic units), shared OTUs at the genus level, shared OTUs at the species level, unique microbial profiles by ethnicity/race, abundance of taxa by ethnicity/race, alpha diversity, beta diversity, transcriptional activity of dominant taxa by ethnic-racial group.

### 2.8. Outcome

The primary outcome investigated in this systematic review was the influence of ethnicity or race on the structure and functional potential of the healthy supragingival dental plaque microbiome.

### 2.9. Data Analysis

A deductive reasoning approach was used to analyze the information obtained, and descriptive statistics were used to summarize and reach consensus on the data of interest by calculating percentages and averages. The proportion and abundance of taxa at the phylum and genus level were represented graphically using the InfoStat statistical package version 2020 (InfoStat Group, FCA, National University of Córdoba, Argentina) [20].

To ensure the reliability of the conclusions and address potential demographic heterogeneity, a subgroup analysis was planned, stratifying the populations into two age groups (under 18 years and 18 years or older) to determine the effect of age on the microbial profiles. Additionally, a qualitative sensitivity analysis was predefined according to the planned temporary exclusion of studies identified as having a low or very low quality of evidence (based on the GRADE) to verify if the primary findings regarding microbiome composition remained robust.

## 3. Results

### 3.1. Demographic and Methodological Characteristics

In the six studies selected for statistical analysis, a total of 474 subjects aged between 4 and 74 years participated; these individuals belonged to six ethnic groups: AA, CA, HA, AS, BM, and AUS. Asians and Australians exhibit a wide ethnic composition; in the case of the AUS group, the population is mainly divided into whites of British origin, non-whites of Asian origin, and Indigenous Torres Strait Islanders. Five of the six selected studies determined microbial identity through 16S-rRNA amplicon sequencing, for which the V3-V4 region was the most evaluated (Table 1). Only one study determined the taxonomic profile of supragingival dental plaque samples through metagenomic sequencing, using the Whole Metagenomic Shotgun Sequencing (WMSS) technique (Table 1).

### 3.2. General Taxonomic Profile in Eubiosis (Core Microbiome)

**Figure 2:**
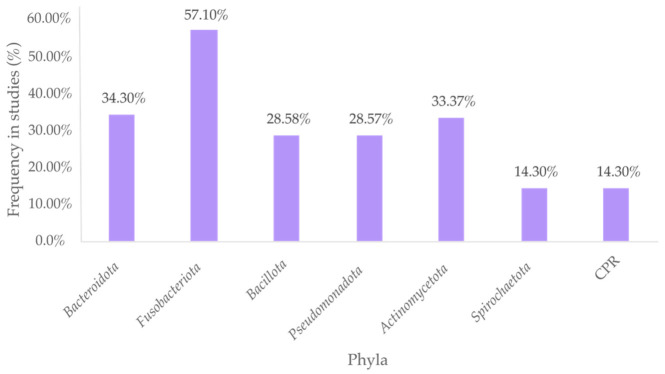
Ubiquitous bacterial phyla in human supragingival dental plaque. More than half of the analyzed studies report *Fusobacteriota* as a prevalent taxon in the human supragingival dental plaque microbiome in eubiosis, regardless of ethnic-racial status.

Statistical analysis determined an average similarity of 91.16% in the dental plaque microbiome composition among Asians. Among the AA, CA, and HA groups, the dental plaque microbiome showed an average homology of 76.6%. At the phylum level, *Fusobacteriota*, followed by *Bacteroidota*, *Actinomycetota*, *Bacillota*, and *Pseudomonadota* demonstrated ubiquity across the evaluated studies (Figure 2). At the level of bacterial genera, *Capnocytophaga* spp., *Leptotrichia* spp., *Fusobacterium* spp., *Streptococcus* spp., and *Neisseria* spp. showed the highest prevalence, being recorded in more than 50% of the analyzed studies (Table 2).

**Figure 3:**
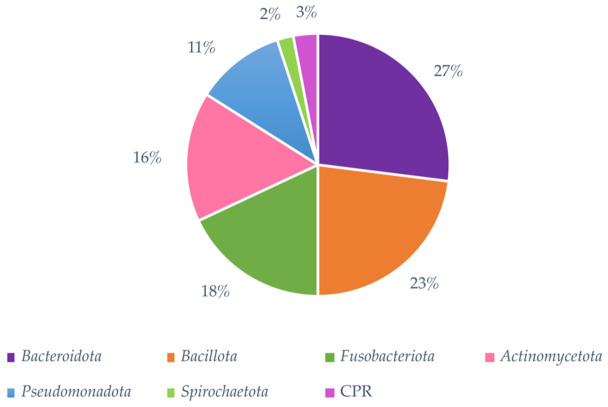
Representative bacterial phyla in human supragingival dental plaque. Descriptive statistical analysis demonstrates that *Bacteroidota*, *Bacillota*, and *Fusobacteriota* are the three most abundant phyla in the supragingival dental plaque microbiome in eubiosis, regardless of ethnic status.

**Figure 4:**
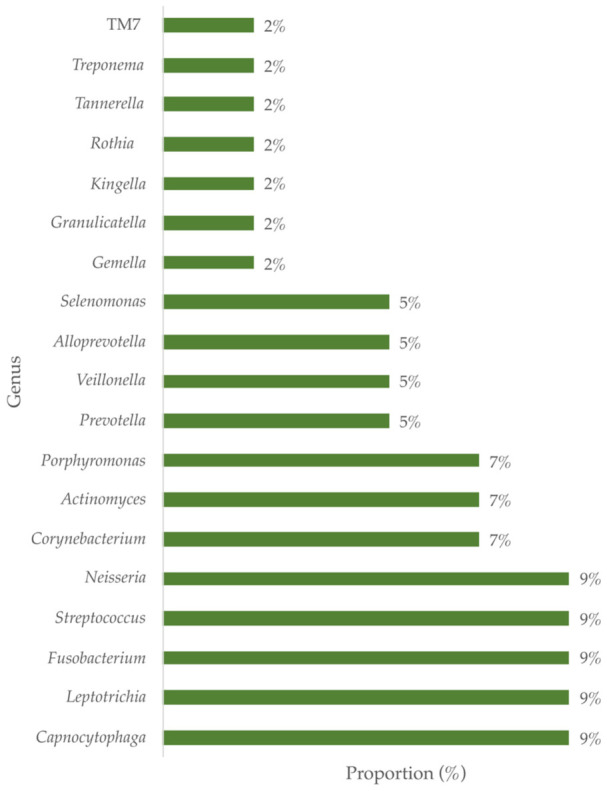
Representative bacterial genera in human supragingival dental plaque. Descriptive analysis demonstrates that these taxa reach an abundance greater than 1% in the microbiome in eubiosis, regardless of ethnic status.

At the species level, only four out of the six analyzed studies reported this taxonomic category, with *Corynebacterium matruchotii* and *Streptococcus gordonii*, being the most reported taxa (Table 2). Regarding the abundance of taxa in supragingival dental plaque in health, the *Bacteroidota*, *Bacillota*, *Fusobacteriota*, *Actinomycetota*, and *Pseudomonadota* phyla demonstrated dominance, representing over 10% of the microbial community, regardless of racial status (Figure 3). At the genus level, *Capnocytophaga* spp., *Corynebacterium* spp., *Streptococcus* spp., *Leptotrichia* spp., *Neisseria* spp., *Actinomyces* spp., *Fusobacterium* spp. and *Porphyromonas* spp., represented more than 5% of the bacterial community of dental plaque, regardless of racial status (Figure 4).

### 3.3. Structural and Ecological Variations by Ethnicity

Based on the results by ethnic group from two primary investigations, the dental plaque microbiome in healthy AA individuals is more diverse than the CA dental plaque microbiome but equals that of HA. It is predominantly composed of the *Fusobacterium* genus and features unique species: *Pedobacter petrophilus* (associated with high levels of *Porphyromonas gingivalis*), *P. gingivalis*, and *Tannerella forsythia*. Compared to CA, AA dental plaque shows a greater abundance of *P. gingivalis* and *Treponema denticola*.

A single study evaluated the functional potential of the supragingival dental plaque microbiome in health; it reported a greater functional abundance of antibiotic resistance genes and genes involved in the modification of glycoconjugates and polysaccharides in the AA group (Table 3).

One study provided information on the dental plaque microbiome of BM; it demonstrated an overrepresentation of the *Capnocytophaga* genus and the unique presence of *Sneathia* spp., when comparing this microbial community with that of AA, CA, and HA. Additionally, it exhibited a high abundance of the *Roseimarinus* and *Treponema* genera, with greater bacterial diversity in this niche compared to AA, CA, and HA, although with low intragroup variability (Table 3).

In CA, two studies present similar results regarding a lower diversity of the dental plaque microbiome in health, with fewer unique bacterial species for this ethnic group compared to AA, HA, and BM. The *Corynebacterium* genus shows enrichment in the supragingival dental plaque of CA in health. Like BM, this microbial community shows high intragroup homology (Table 3).

Based on results from two primary investigations, the structure of HA dental plaque in health is comparable to that of BM and AA. In fact, HA dental plaque in eubiosis shows a high prevalence of the *Capnocytophaga* genus with a high abundance of periodontal pathogens like *Filifactor alocis*, *P. gingivalis*, and *T. forsythia* when compared to CA. One study reported a total of 310 unique bacterial species for this racial group, such as an unclassified Micrococcales bacterium and *Megasphaera* sp. Regarding the diversity of this bacterial community, the data are contradictory: one study shows significantly lower bacterial diversity than AA but similar to CA, while another study shows species enrichment in HA similar to that exhibited by AA for this ecological niche, with specific taxa present in situations with a high dental plaque index. Regarding the functional potential of dental plaque in HA, one study reveals a lower abundance of genes encoding carbohydrate metabolism enzymes compared to AA (Table 3).

Among Asian ethnic groups, a total of three primary studies were obtained on the structure of healthy dental plaque. Based on this body of evidence, Pseudomonadota, Bacteroidota, Fusobacteriota, and Bacillota demonstrate dominance in this racial group (Table 3), with a high prevalence of *Streptococcus* spp., *Campylobacter* spp., *Prevotella* spp., *Fusobacterium* spp., *Leptotrichia* spp., *Neisseria* spp., *Capnocytophaga* spp., *Kingella* spp., *Actinomyces* spp., *Bacteroides* spp., *Centipeda* spp., and *Paracoccus* spp., while *Aggregatibacter* spp., *Enterococcus* spp., *Bacillus* spp., *Selenomonas* spp., *Oribacterium* spp., *Dialister* spp., *Olsenella* spp., *Streptococcus* spp., *Neisseria* spp., *Simonsiella* spp., *Capnocytophaga* spp., *Kingella* spp., and *Pseudopropionibacterium* spp. stand out in colonization abundance. At the species level, *Capnocytophaga gingivalis*, *S. oralis* subsp. *dentisani*, and *Kingella denitrificans* demonstrated a key role as dental health biomarkers. At the functional level, PICRUSt analysis did not show significant differences in activated metabolic pathways among Asian ethnic groups.

A single study describes the microbial structure of the dental plaque of native Australian subjects, which shows lower diversity compared to the dental plaque of non-native AUS, with an overrepresentation of four genera: *Neisseria*, *Kingella*, *Haemophilus*, and *Rothia*, and an elevated abundance of *Streptococcus* spp. and *Corynebacterium* spp. in the presence of a diet high in sugar and fat (Table 3).

**Figure 5:**
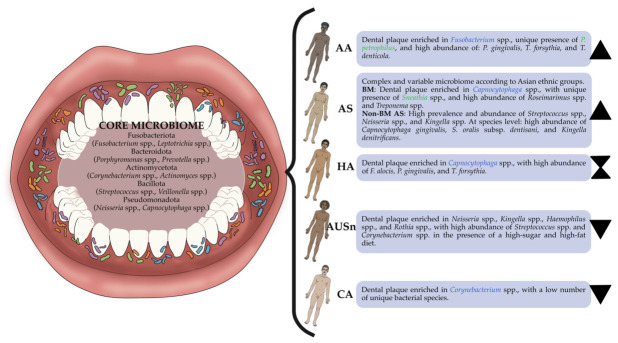
Summary of the core microbiome and differential taxonomic signatures by ethnicity. The left side illustrates the basal bacterial architecture (core microbiome) associated with dental health, regardless of ethnic origin. The right side displays the distinctive bacterial profiles and structural variations in dental plaque according to ancestry. The dental plaque of AA and AS is more biodiverse compared to that of CA. Australian-born individuals (AUSn) present a less diverse microbiome than those born overseas. Regarding the diversity of the dental plaque microbiome in HA, the evidence remains inconclusive. Legend: (🟦) Prevalent microbial groups; (🟩) Unique microbial groups; (⧗) Inconclusive diversity; (▲) High diversity; (▼) Low diversity.

To date, no studies have been published comparing the structure of human dental plaque between Asian populations and AA, CA, HA, or AUS groups, nor between AUS and AA, CA, HA, or AS groups. A comprehensive summary of the core microbiome and the differential taxonomic signatures across the evaluated ethnic groups is presented in Figure 5.

### 3.4. Subgroup and Sensitivity Analysis

#### 3.4.1. Impact of Age

To evaluate the impact of age on the oral microbiome, a subgroup analysis was conducted, stratifying the evidence into two age groups (populations < 18 years: childhood and adolescence, and >18 years: adulthood). The cross-sectional comparison of these groups revealed notable temporal stability within the core microbiome; fundamental taxa such as *Streptococcus* (Bacillota), *Neisseria* (Pseudomonadota), *Fusobacterium* (Fusobacteriota), and *Capnocytophaga* (Bacteroidota) demonstrated structural dominance in a state of eubiosis, regardless of the evaluated age group.

Even more significantly, when directly contrasting African American (AA), Caucasian (CA), and Hispanic American (HA) populations with cohorts in notably different age groups (4–6 years vs. 21–75 years), it became evident that ancestry-driven taxonomic signatures persist over time. Specific microbial profiles, such as the lower diversity and marked dominance of *Corynebacterium* in CA, as well as the higher bacterial diversity and enrichment of periodontal pathogenic species in eubiosis within AA and HA populations, remain consistent from an early age into adulthood/senescence (Table 4).

#### 3.4.2. Concordance According to Sequencing Technology

When stratifying the results by the sequencing technology employed, a notable ecological concordance was observed between 16S rRNA amplicon sequencing and whole-metagenome shotgun sequencing (WMSS). Evaluating the same three populations (African Americans, Caucasians, and Hispanic Americans), both technologies confirmed identical diversity patterns: Caucasians (CA) systematically exhibit the lowest bacterial richness, while the African American (AA) population maintains the highest diversity (Table 5).

#### 3.4.3. Sensitivity Analysis

To ensure the robustness of the findings, a sensitivity analysis was performed based on the critical appraisal of the certainty of evidence using GRADE (Supplementary Table S3). The overall analysis determined that 66.67% (*n* = 4) of the primary studies presented moderate to high-quality evidence. By temporarily excluding literature with low or very low levels of evidence (Wang et al. (2024) [25] and Premaraj et al. (2020) [21]), the core microbial profile remained constant, with the consistently prominent expression of the genera *Streptococcus*, *Neisseria*, and *Capnocytophaga* (Supplementary Table S4). However, the exclusion of these two studies removes the primary data sources evaluating the African American, Caucasian, Hispanic American, and Burmese populations. Consequently, the comparative analysis of peripheral taxonomic signatures by ethnicity in this sensitivity model is restricted strictly to the remaining Asian and Australian cohorts.

## 4. Discussion

The primary objective of this systematic review was to determine whether ethnicity influences the composition and function of the supragingival dental plaque microbiome in a state of eubiosis. After analyzing the six included studies, the findings suggest a direct influence of ethnicity on oral ecology; this phenomenon is reflected in the abundance of specific taxa, even in populations free from clinical pathology. Although a shared core microbiome was identified across the compared ethnic groups, this review revealed a distinctive taxonomic configuration characterized by the enrichment of *Fusobacterium* spp. in AA, the dominance of *Corynebacterium* spp. in CA, and the identification of *Sneathia* spp. as an exclusive taxon in the BM population. These findings reinforce the hypothesis that the microbial profile in health is not a universal standard, but rather a spectrum of ecological balances associated with host ethnic-racial factors.

Historically, pioneering studies on the composition of the oral microbiome, such as that by Nasidze et al. [10], suggested a lack of distinct geographic signatures, as they observed greater variation among individuals from the same area than between different geographic regions. However, our results regarding supragingival plaque challenge this notion of uniformity. Interestingly, that same research reported the exclusive presence of the genus *Enterobacter* in samples from the Congo and its absence in other regions. Although the authors did not ascribe significant importance to this finding, we argue that it represents one of the earliest pieces of evidence for specific taxonomic signatures within a healthy ethnic group. Furthermore, this discrepancy may be attributed to the nature of the studied ecological niche: saliva (the focus of many previous studies) is a dynamic fluid, whereas dental plaque is a highly structured biofilm that adheres to the tooth surface [4,6]. In this sessile microenvironment, host genetics and the innate immune response may exert a stricter selective pressure on bacterial colonization compared to the salivary environment.

Although the primary focus of our hypothesis centers on ethnic variability, it is crucial to highlight that these variations occur upon a shared structural foundation. In line with the “core microbiome” hypothesis proposed by Zaura et al. [6], we emphasize the ubiquitous presence of key genera such as *Corynebacterium* and *Streptococcus*, regardless of ethnicity. This finding aligns seamlessly with the biological framework of the “hedgehog” architectural model described by Mark Welch et al. [4]. The constant presence of *Corynebacterium* across all ethnic cohorts suggests that this genus serves a fundamental engineering role, frequently anchoring to the dental surface alongside *Actinomyces* spp. [4]. Upon this foundation, streptococci organize into “corncob” structures, establishing the three-dimensional physical integrity necessary for biofilm homeostasis [4,27]. Based on the existing literature and our current findings, we propose that “the architecture is universal”—a prerequisite for biofilm survival—while the composition of peripheral species varies according to the individual’s ancestry.

With respect to structural and ecological variations, our results corroborate those of Mason et al. [12] and Wang et al. [28], who highlighted significant disparities between AA and CA cohorts within a shared geographical environment, pointing to a strong influence of host genotype. However, evidence from Ma et al. [29] and Demehri et al. [30] introduces a critical nuance: in highly diverse or urbanized populations, environmental factors such as diet strongly modulate microbiome diversity. Therefore, we deduce that while the ethnic signature is robust in conserved communities, environmental homogenization (e.g., a “Western lifestyle”) acts as a selective pressure that masks, rather than replaces, genetic variations. This refines our hypothesis: ethnicity establishes a baseline biological susceptibility that the environment can exacerbate or mitigate, reinforcing the need for personalized dentistry to integrate both ancestry and lifestyle.

Although our study focuses on identifying baseline structural profiles, it is pertinent to highlight the work of Wang et al. [28], who investigated the microbiological factors underlying disparities in periodontal health. Their research revealed that periodontal risk depends not only on the relative abundance of periodontopathogenic bacteria but also on their specific genetic variants. Specifically, they found that *P. gingivalis* is not only more prevalent in AA and HA cohorts, but is also predominantly present as its *fimA* II and *fimA* Ib genotypes, both of which are known for their high capacity for adhesion and tissue invasion [31]. Consequently, AA and HA individuals may exhibit a greater predisposition to mount more severe inflammatory responses compared to CA individuals. Conversely, Wang et al. [28] identified the presence of *Streptococcus cristatus* as a protective marker in CA, as it represses the expression of fimbriae (*fimA*) and subsequent colonization by *P. gingivalis*. Therefore, its identification within the microbiome can be interpreted as a health biomarker, the absence or reduction in which in AA and HA populations could partially explain their higher susceptibility to more aggressive forms of periodontal disease.

Concerning species richness, direct comparisons across the existing literature are challenging and often precluded by methodological and demographic heterogeneities. For instance, while our findings suggest greater microbial diversity in the AA cohort, previous studies such as Mason et al. [12] reported lower diversity indices in this demographic compared to CA individuals. A plausible explanation for these divergent observations, beyond the specific sequencing methodologies employed, lies in the distinct characteristics of the studied populations, particularly their degree of urbanization and dietary habits. Indeed, Ma et al. [29] demonstrated that individuals from urban environments exhibited greater microbial diversity than their rural counterparts. This concept is further corroborated by Nath et al. [24] in Australia, who observed that native-born subjects exhibited significantly lower diversity than immigrants, attributing this ecological impoverishment to a diet rich in sugar and fat. Nevertheless, as recently highlighted by Manghi et al. [32] in a large-scale metagenomic analysis, while environmental factors and lifestyle significantly modulate the oral community, certain microbial signatures remain remarkably resilient across different populations. Collectively, this suggests that microbial diversity is a dynamic trait shaped by lifestyle and dietary patterns, but one that does not completely overshadow the underlying ancestry-driven biological core.

It is important to consider certain modulating factors of the human oral microbiome. However, the literature demonstrates that a child’s oral microbiome stabilizes rapidly, reaching an adult-like composition by the end of primary dentition (between 3 and 5 years of age) [16]. This biological stability justifies the inclusion of pediatric cohorts in our review. More importantly, it is strongly supported by our age stratification analysis (Section 3.4.1), which confirms that ancestry-driven taxonomic signatures are not late-stage ecological shifts, but rather early-colonization events that transcend age. By contrasting populations under and over 18 years of age, we observed that specific profiles—such as the early enrichment of certain periodontal pathogens in AA and HA cohorts, or the low diversity and marked dominance of Corynebacterium in CA—are established in early childhood and persist into adulthood.

Importantly, the interpretation of these results must account for methodological heterogeneity. There is a notable disparity in sample sizes, ranging from robust cohorts to very small groups, which constrains the statistical power to define distinctive ethnic-specific microbial profiles. Furthermore, variations in sequencing platforms—ranging from 16S rRNA gene amplicon sequencing to WMSS—introduce technical variability that can complicate direct comparisons. However, following a comparative analysis, a strong ecological concordance between the employed methodologies was observed. Despite technical differences in depth and resolution, both methodologies confirm similar diversity patterns driven by ethno-racial factors. This highlights a lower bacterial richness in the CA cohort and a higher diversity with the enrichment of specific pathogens in AA populations, thereby reinforcing the validity of these signatures regardless of the different sequencing strategies utilized.

Moreover, the certainty of these findings must be rigorously weighed against the quality of the available evidence. By applying the GRADE assessment tool to conduct a rigorous sensitivity analysis, we observed that while the universal core microbiome (dominated by *Streptococcus*, *Neisseria*, and *Capnocytophaga*) is robustly supported by moderate- to high-quality evidence, specific ethnic variations rely heavily on lower-certainty data. The exclusion of studies with low evidence levels restricts the ethno-racial comparative landscape, highlighting a significant limitation in the current literature. Therefore, at this stage, we can confidently support the presence of a core microbiome; however, it is imperative to conduct future primary studies with more rigorous methodological designs. These studies should encompass better-defined populations across significant geographic regions to definitively validate the peripheral characteristics of the microbiome associated with the unique ethno-racial factors of each individual.

### Clinical Implications and Future Directions

The findings of this review support a transition toward dental management grounded in the biological and ancestral profile of the patient. Traditionally, preventive programs have been based on behavioral (hygiene, diet) or socioeconomic risk factors. However, evidence of greater biological susceptibility in AA and HA cohorts—associated with the presence of virulent genotypes of *P. gingivalis* (*fimA* II/Ib) and the absence of protective factors such as *S. cristatus*—suggests that risk stratification incorporating ancestry may be warranted.

Consequently, these findings suggest that differentiated periodontal screening protocols could be clinically beneficial. Future longitudinal studies are warranted to evaluate whether individuals from specific ancestral cohorts (such as AA and HA) might benefit from early microbiological monitoring and adjusted maintenance intervals—potentially more frequent than the standard 6-month recall—even in the absence of apparent clinical disease. Recognizing ancestry as a potential biological risk indicator, alongside established social determinants, could eventually enable healthcare systems to design more personalized preventive interventions for populations that are potentially more vulnerable to dysbiosis and severe periodontal disease.

## 5. Conclusions

Our findings suggest a direct influence of ethnicity on the structure and function of the supragingival dental plaque microbiome in eubiosis. Although there is a universally shared architecture that is robustly supported by moderate to high-quality evidence, host ancestry defines distinctive taxonomic profiles among peripheral species, such as the enrichment of *Fusobacterium* spp. in AA cohorts and the unique presence of *Sneathia* spp. in BM populations. Furthermore, our analysis indicates that these ancestry-driven signatures are established in early childhood and transcend age. However, this variability is not static; environmental factors—such as diet and urbanization—can strongly modulate ecological diversity, potentially attenuating or exacerbating underlying genetic predispositions. Given that the current evidence for specific ethnic variations relies on lower-certainty data, it is imperative to conduct future primary studies with rigorous methodological designs and broader geographic representation to definitively validate these observations.

From a clinical perspective, our findings challenge the traditional paradigm of standardized dental treatment in the management of periodontal diseases. Certain ethnic cohorts, specifically AA and HA, may exist in a baseline state of health that is under constant ecological challenge, given the ethnicity-associated carriage of bacterial genotypes with greater virulence.

## Figures and Tables

**Table 1:** Summary of demographic characteristics, study design, and methodology of the included studies.

Authors and Publication Year	Study Title	Compared/Studied Ethnic Groups	Geographic Region	No. of Patients	Age Range	Health Condition	Methodology	Evidence Analysis (GRADE)
Premaraj et al. [21]	Ethnic variation in oral microbiota	African Americans (AA), Burmese (BM), Caucasian (CA), Hispanic (HA)	USA (North America)	96	6–11 years	Healthy children, classified into high/low plaque index and high/low DMFT index groups.	16S rRNA gene sequencing targeting the V3-V4 region.	Low
Zhang et al. [22]	Ethnicity-based analysis of supragingival plaque composition in healthy subjects without caries	Zhuang and Han (From China)	China (Asia)	96	4–5 years	Healthy children free of caries and periodontal diseases.	16S rRNA sequencing targeting the V4 region.	Moderate
Luo et al. [23]	Identifying the oral microbiome of adolescents with and without dental fluorosis based on full-length 16S rRNA gene sequencing	Chinese. Miao ethnic group and other ethnic groups (Chuanqing, Han, Yi)	China (Asia)	23	9–18 years	Healthy (H) and healthy Miao (Hm) children.	High-throughput full-length 16S rRNA gene sequencing.	High
Nath et al. [24]	Characterising healthy Australian oral microbiomes for ‘super donor’ selection	Australian residents: Australian-born and Overseas-born (multi-ethnic	Australia	93	Older than 18	Caries-free patients, with no periodontal pockets and BoP < 20%.	16S amplicon sequencing.	High
Wang et al. [25]	Diversity and Characteristics of the Oral Microbiome Associated with Self-Reported Ancestral/Ethnic Groups	African Americans (AA), Caucasian Americans (CA), Hispanic Americans (HA)	USA (North America)	161	21–75 years	Healthy adults; periodontal health or biofilm-induced gingivitis on an intact periodontium.	WMSS performed using Illumina NovaSeq (150 bp paired-end).	Very Low
Miao et al. [26]	Supragingival microbial profiles in caries-free and caries-active adolescents treated with fixed orthodontics	Unique ethnic group (Han)	China (Asia)	5	12 years	Healthy children undergoing orthodontic treatment.	High-throughput 16S rRNA gene sequencing targeting the V3–V4 region.	Moderate

BoP: Bleeding on Probing; WMSS: Whole Metagenome Shotgun Sequencing; DMFT: Decayed, Missing and Filled Teeth.

**Table 2:** Distribution of bacterial taxa at the Phylum, Genus, and Species levels across all analyzed studies.

Genus	Phylum	Frequency in Studies (%)	Reported Species	Frequency in Studies (%)
*Capnocytophaga* spp.	Bacteroidota	57.1	*Corynebacterium matruchotii*	50
*Leptotrichia* spp.	Fusobacteriota	57.1	*Tannerella forsythia*	25
*Fusobacterium* spp.	Fusobacteriota	57.1	*Fusobacterium nucleatum*	25
*Streptococcus* spp.	Bacillota	57.1	*Streptococcus gordonii*	50
*Neisseria* spp.	Pseudomonadota	57.1	*Streptococcus cristatus*	25
*Corynebacterium* spp.	Actinomycetota	42.9	*Porphyromonas gingivalis*	25
*Prevotella* spp.	Bacteroidota	28.6	*Treponema denticola*	25
*Veillonella* spp.	Bacillota	28.6	*Filifactor alocis*	25
*Actinomyces* spp.	Actinomycetota	42.9	*Streptococcus mitis*	25
*Porphyromonas* spp.	Bacteroidota	42.9	*Streptococcus oralis*	25
*Alloprevotella* spp.	Bacteroidota	28.6	*Streptococcus sanguinis*	50
*Gemella* spp.	Bacillota	14.3	*Veillonella parvula*	25
*Granulicatella* spp.	Bacillota	14.3	*Capnocytophaga granulosa*	25
*Kingella* spp.	Pseudomonadota	14.3	*Neisseria mucosa*	25
*Rothia* spp.	Actinomycetota	14.3		
*Tannerella* spp.	Bacteroidota	14.3	*Haemophylus parainfluenzae*	25
*Treponema* spp.	Spirochaetota	14.3		
*Selenomonas* spp.	Bacillota	28.6	*Veillonella dispar*	25
TM7	CPR	14.3		
*Cardiobacterium* spp.	Pseudomonadota	14.3		

CPR: Candidate Phyla Radiation.

**Table 3:** Reported composition, abundance, diversity, and transcriptional activity according to the studied ethnic groups.

Ethnic Group	Country of Study	Composition (Dominant/Key Taxa)	Abundance (Significant Differences)	Alpha/Beta Diversity	Transcriptional Activity	Author
AA	USA	*Fusobacterium* as the dominant genus.	High relative abundance of *Prevotella* and *Leptotrichia*.	Greater diversity than Caucasians, but no significant difference in richness compared to HA.	Not reported.	Premaraj et al. [21]
	USA	Unique species found: *Pedobacter petrophilus* (associated with high levels of *P. gingivalis*). Presence of *P. gingivalis* and *T. forsythia*.	Higher abundance of *P. gingivalis* and *T. denticola* compared to CA.	Greater diversity: non-redundant genes and species are significantly higher than in CA and HA.	High functional potential: Greater abundance of genes for glycoconjugate and polysaccharide modification.	Wang et al. [25]
BM	USA	*Capnocytophaga* as the dominant genus. Unique taxon: *Sneathia*.	17 unique OTUs found in this group. *Roseimarinus* and *Treponema* are more dominant.	Highest diversity: Bacterial richness is significantly greater than in the other three ethnic groups (AA, CA, and HA). High intragroup similarity.	Not reported.	Premaraj et al. [21]
CA	USA	*Corynebacterium* as the dominant genus.	Dominant OTUs: *Prevotella* and Lachnospiraceae.	Lowest diversity: Richness significantly lower than any other studied ethnicity (AA, BM, and HA). High intragroup similarity.	Not reported.	Premaraj et al. [21]
	USA	Lower presence of unique species compared to AA and HA.	Lower abundance of periodontal pathogens (*P. gingivalis*, *T. denticola*) compared to AA and HA.	Lower diversity of species and non-redundant genes compared to AA.	Lower functional potential for antibiotic resistance compared to AA.	Wang et al. [25]
HA	USA	*Capnocytophaga* as the dominant genus.	Specific OTUs: *Treponema* and *Marinisporobacter*.	No significant difference in richness compared to AA. Intragroup variation similar to intergroup variation.	Not reported.	Premaraj et al. [21]
	USA	310 unique species identified. Presence of *Micrococcales* bacterium and *Megasphaera* sp.	Higher abundance of *F. alocis* and *T. forsythia* compared to CA. Abundance of *P. gingivalis* is higher than in CA.	Diversity is significantly lower than in AA, but no significant difference with CA.	Lower abundance of carbohydrate-active enzyme genes compared to AA.	Wang et al. [25]
AS Multiethnic (Zhuang, Han, Miao, among others)	China (Asia)	Dominant phyla: *Proteobacteria, Firmicutes.* Genera: *Streptococcus*, *Neisseria*.	Higher abundance of *Aggregatibacter*, *Enterococcus*, and *Bacillus*. Higher abundance of Bacillaceae and Enterococcaceae families.	No significant difference in Alpha or Beta indices.	Functional prediction (PICRUSt): No significant differences in metabolic pathways.	Zhang et al. [22]
	China (Asia)	Dominant genera in the caries-free group: *Leptotrichia* (22.9%), *Fusobacterium* (8.8%), *Prevotella* 7 (7.6%), *Campylobacter* (6.0%), and *Streptococcus* (non-mutans) (5.95%).	Health biomarkers: Significant enrichment of *Selenomonas* 3, *Oribacterium*, *Dialister*, and *Olsenella* in healthy adolescents undergoing orthodontic treatment. Absence of *S. mutans* and lower abundance of *Neisseria* compared to diseased groups.	Stable core microbiome: Although Alpha diversity tends to be slightly higher in the caries-free group than in the caries-active group, there is no significant statistical difference, indicating a stable and resilient community despite orthodontic treatment.	Not reported.	Miao et al. [26]
	China (Asia)	Dominant phyla: Proteobacteria, Firmicutes. Dominant genera: *Streptococcus* (22.7%), *Neisseria* (20.5%).	Higher relative abundance of the genus *Simonsiella*.	No significant difference in Alpha and Beta diversity compared to Zhuang.	Not reported.	Zhang et al. [22]
	China (Asia)	Healthy Miao (Hm) microbiome: *Capnocytophaga*, *Kingella*, *Actinomyces*, *Leptotrichia*. Unique genera in healthy individuals: *Bacteroides*, *Centipeda*, *Paracoccus*.	Health biomarkers: Significantly higher abundance of *Capnocytophaga gingivalis* and *Kingella denitrificans*. *Corynebacterium* showed a positive correlation in healthy interaction networks.	Higher diversity: The healthy Miao group presented a significantly higher Shannon index and a distinct community structure (Beta diversity) compared to the fluorosis group.	Functional prediction (PICRUSt2): No significant differences.	Luo et al. [23]
	China (Asia)	General healthy microbiome: *Streptococcus*, *Neisseria*, *Aggregatibacter*. Key species: *Streptococcus oralis* subsp. *dentisani*.	Health biomarkers: Higher abundance of *Pseudopropionibacterium* and *S. oralis* subsp. *dentisani* (strain with probiotic/anti-acid potential). Exclusive presence of *Bacteroides* and *Paracoccus* in healthy groups.	Stability: No significant differences in Alpha and Beta diversity compared to the diseased group, suggesting a resilient core microbiome.	Functional prediction (PICRUSt2): No significant differences.	Luo et al. [23]
AUS	Australia	Australian-born individuals exhibited a higher abundance of *Neisseria*, *Haemophilus*, *Kingella*, and *Rothia*.	Greater association with a diet high in sugar/fats. *Streptococcus* and *Corynebacterium* are prevalent.	Lower diversity compared to overseas-born individuals.	Not reported.	Nath et al. [24]
	Australia	Overseas-born individuals exhibited a higher abundance of *Alloprevotella*, Lachnospiraceae, and *Parvimonas*.	Higher abundance of *Prevotella* and *Alloprevotella* correlated with birthplace.	Greater diversity: Alpha and Beta diversity are significantly higher than in Australian-born individuals.	Not reported.	Nath et al. [24]

OTU: Operational Taxonomic Unit; PICRUSt: Phylogenetic Investigation of Communities by Reconstruction of Unobserved States.

**Table 4:** Comparison of the Core Microbiome by Age Group and Ethnicity in a State of Eubiosis.

Ethnic Group	Under 18 Years (<18)	18 Years and Older (≥18)
African Americans (AA)	Dominance of *Fusobacterium*, with high abundance of *Prevotella* and *Leptotrichia*.	High abundance of *Porphyromonas* (*P. gingivalis*) and *Treponema* (*T. denticola*). Exclusive presence of *Pedobacter*.
Hispanic Americans (HA)	Dominance of *Capnocytophaga*, with specific presence of *Treponema* and *Marinisporobacter*.	High abundance of *Filifactor* (*F. alocis*) and *Tannerella* (*T. forsythia*).
Caucasians (CA)	Marked dominance of *Corynebacterium*.	Dominance of *Corynebacterium*. Lower presence of periodontal pathogens compared to AA and HA.
Asians (AS) (Han, Zhuang, Miao)	Dominance of *Streptococcus*, *Neisseria*, *Leptotrichia*, *Capnocytophaga*, *Actinomyces*, and *Kingella*.	No data available for comparison
Burmese (BM)	Dominance of *Capnocytophaga*, with exclusive presence of *Sneathia*.	No data available for comparison
Australians (AUS)	No data available for comparison	Natives: Dominance of *Neisseria*, *Streptococcus*, *Corynebacterium*, and *Haemophilus*. Immigrants: *Alloprevotella*, *Parvimonas*, and *Prevotella*.

Note: This table contrasts the dominant taxonomic profiles and specific microbial signatures among different ethno-racial groups, stratified by age (<18 years and ≥18 years), highlighting the temporal stability of ancestry-driven microbial signatures.

**Table 5:** Ecological Concordance of the Oral Microbiome According to Sequencing Technology.

Ethnic Group	16S rRNA Sequencing (Amplicon)	Metagenomic Sequencing (WMSS)	Comparative Analysis (Concordance)
	Premaraj T.S, et al., 2020 [21]	Wang Q, et al., 2024 [25]	
AA	Diversity: High. Taxonomic Profile: Abundance of *Fusobacterium* (OTU_3) and *Pseudopropionibacterium.*	Diversity: Highest (in non-redundant genes and species). Taxonomic Profile: Higher abundance of pathogens (*P. gingivalis*, *T. denticola*) and unique species *Pedobacter petrophilus.*	High concordance. Both methods confirm that the AA microbiome possesses higher diversity/richness and a marked inclination towards ecosystems rich in Gram-negative and anaerobic bacteria.
CA	Diversity: Lowest of all studied ethnicities. Taxonomic Profile: Marked dominance of *Corynebacterium* (OTU_10) and *Prevotella* (OTU_101).	Diversity: Low (fewer number of species and genes compared to AA). Taxonomic Profile: Lower presence of periodontal pathogens.	High concordance. WMSS technology confirms the 16S finding: the plaque ecosystem in healthy Caucasians is less diverse and ecologically more conservative, dominated by basal taxa.
HA	Diversity: Similar to AA. Taxonomic Profile: Presence of specific taxa such as *Treponema* and *Marinisporobacter.*	Diversity: Lower than AA, but with a consolidated risk profile. Taxonomic Profile: High abundance of *F. alocis* and *T. forsythia*. 310 unique species detected.	Complementary concordance. Both confirm a complex ecosystem with exclusive bacterial niches. WMSS was able to identify exact species that 16S only grouped at the OTU level.

Note: Comparative analysis of taxonomic profiles, diversity metrics, and specific findings obtained via 16S rRNA amplicon sequencing versus Whole-Metagenome Shotgun Sequencing (WMSS) across AA, CA and HA populations.

## Data Availability

The protocol for this systematic review, titled “Structure and Function of the Dental Plaque Microbiome in Eubiosis: A Systematic Review on Ethnic Influences”, was pre-registered and is publicly available on the Open Science Framework (OSF) platform (registered on 29 January 2026; https://doi.org/10.17605/OSF.IO/5Y4W7; accessed on 29 March 2026). Additional data presented in this study are available on request from the corresponding author.

## Supplementary Materials

The following supporting information can be downloaded at: https://www.mdpi.com/article/10.3390/microorganisms14051095/s1, Supplementary Table S1: Traceability of the search strategy and study selection process across consulted databases; Table S2: Excluded studies and their specific reasons for exclusion after full-text review [12,24,28,29,30,33,34,35,36,37,38,39,40,41,42,43,44,45,46]; Table S3: GRADE Certainty of Evidence Assessment for the Included Studies; Table S4: Methodological characteristics, certainty of evidence (GRADE), and core microbiome profiles of the included studies.

## Abbreviations

The following abbreviations are used in this manuscript:

AA	African Americans
AS	Asians
AUS	Australians
AUSn	Australian-born individuals
BM	Burmese
BoP	Bleeding on Probing
CA	Caucasians
DMFT	Decayed, Missing and Filled Teeth
HA	Hispanic Americans
OSF	Open Science Framework
OTU	Operational Taxonomic Unit
PI	Plaque Index
PICRUSt	Phylogenetic Investigation of Communities by Reconstruction of Unobserved States
PPD	Probing Pocket Depth
PRISMA	Preferred Reporting Items for Systematic Reviews and Meta-Analyses
WMSS	Whole Metagenome Shotgun Sequencing
