# Supplementary material for: Structure and Function of the Dental Plaque Microbiome in Eubiosis: A Systematic Review of Ethnic-Racial Influences
Source: Microorganisms. 2026 May 12;14(5):1095. doi: 10.3390/microorganisms14051095 (PMC13209762; doi:10.3390/microorganisms14051095)
Supplement: Supplementary file 1 [file microorganisms-14-01095-s001.zip › microorganisms-4259695-supplementary.pdf]

Structure and Function of the Dental Plaque Microbiome in Eubiosis: A Systematic Review of Ethnic-Racial Influences

Edisson Ronaldo Duran Yunga 1, and María de Lourdes Rodriguez Coyago* 1,2,3

1 Facultad de Odontología, Universidad de Cuenca, Cuenca 010107, Ecuador;

2Departamento de Microbiología y Diagnóstico, Facultad de Odontología, Universidad de Cuenca, Cuenca - Ecuador

3. Grupo de Investigación en Rehabilitación Oral (GIRO), Facultad de Odontología, Universidad de Cuenca, Cuenca - Ecuador.

Supplementary text

Table S1. Traceability of the search strategy and study selection process across consulted databases.

PRISMA 2020 category	Subcategory	Number of Records (n)	Traceability (Search String / Action)
Identification	PubMed	62	("oral microbiome"[Title/Abstract] OR "oral microbiota"[Title/Abstract]) AND ("ethnic groups"[MeSH Terms] OR "ethnicity"[Title/Abstract] OR "race"[Title/Abstract])
	ScienceDirect	530	"dental plaque" AND (composition OR biogeography) AND (ethnicity OR race) AND health
	Scopus	116	("oral microbiome" OR "oral microbiota" OR "dental plaque") AND ("ethnicity" OR "race" OR "ethnic groups" OR "ancestry" OR "population group") AND ("health")
	SciELO	5	"dental plaque" AND structure OR composition AND ethnicy OR race
	Total of results	713	Sum of all sources.

	Total before deduplication	298	Sum of all sources excluding non-open access articles.
	Removed records (duplicates)	298 - 26 = 272	Zotero results.
Screening	Records screened (Title/Abstract)	272	Unique records.
	Records excluded (Title/Abstract)	247	Documentation in the screening log.
Eligibility	Full-text articles retrieved for full evaluation	25	Retained records.
	Exclusion after complete review	25-19=6	Reports excluded:<br>Sample type =16<br>No health specification= 2<br>Study design= 1
Included	Studies included in review	6	Total of included studies.

MeSH: Medical Subject Headings.

Table S2. Excluded studies and their specific reasons for exclusion after full-text review.

Author and publication year	Study title	Reason for exclusion	Specific detail
Ghannoum et al. 2010	Characterization of the Oral Fungal Microbiome (Mycobiome) in Healthy Individuals	Sample type / Domain	The study used oral rinse samples (not plaque) and focused on the mycobiome (fungi) rather than the bacteriome.
Soncini et al. 2010	Oral microbiota of children in a school-based dental clinic	Health condition / Objective	The main objective was to compare caries status (active vs. caries-free) and not ethnic groups in eubiosis.
Contreras et al. 2010	The bacterial microbiota in the oral mucosa of rural Amerindians	Sample type	It analyzed the microbiota of the oral mucosa and dorsum of the tongue, niches distinct from the tooth.
Mason et al. 2013	Deep sequencing identifies ethnicity-specific bacterial	Sample type	It used subgingival plaque and saliva samples, not supragingival plaque.

	signatures in the oral microbiome		
Moon and Lee 2016	Probing the diversity of healthy oral microbiome with bioinformatics approaches	Sample type	The study used saliva samples (evaluating different DNA extraction methods), not dental plaque.
Renson et al. 2019	Sociodemographic variation in the oral microbiome	Sample type	The study (HELMi cohort) used saliva samples, not dental plaque.
Yang et al. 2019	Racial differences in the oral microbiome: Data from low-income populations of African ancestry and European ancestry	Sample type	Samples were collected via oral rinse, which analyzes saliva and mucosa, not structured supragingival biofilm.
Inquimbert et al. 2019	Microbiota of interdental space of adolescents according to Risk of Caries: A cross-sectional study protocol	Study design	It is a study protocol, presenting no results. Furthermore, it focuses on interdental plaque and caries risk.
Liu et al. 2021	Oral Microbiota of Children Is Conserved across Han, Tibetan and Hui Groups and Is Correlated with Diet and Gut Microbiota	Sample type	Although it compared ethnicities, the study used saliva samples.
Dong et al. 2021	Comparative Study of Oral Bacteria and Fungi Microbiota in Tibetan and Chinese Han Living at Different Altitude	Sample type	Sample collection consisted of saliva (2 mL, unstimulated), not supragingival dental plaque.
Ma et al. 2022	Comparison of the Oral Microbiota Structure among People from the Same Ethnic Group Living in Different Environments	Health condition / Study design	The study did not differentiate between a healthy and a diseased population.
Altayb et al. 2022	Study of oral microbiota diversity among groups of families originally from different countries	Sample type	Sample collection was performed exclusively using saliva.
Marcano-Ruiz et al. 2023	Oral microbiota, co-evolution, and implications for health and disease: The case of indigenous peoples	Study design	It is a review and opinion article, not a primary observational study with original cross-sectional data.

Wang et al. 2023	Identification of microbiological factors associated with periodontal health disparities	Study objective / Sample type	It focuses on the specific proportion of two species (*P. gingivalis* and *S. cristatus*) in subgingival plaque, not on the complete taxonomic characterization of the supragingival community in eubiosis.
Ogbanga et al. 2023	The Oral Microbiome for Geographic Origin: An Italian Study	Sample type	The study uses saliva samples to determine geographic origin.
Demehri et al. 2024	Supragingival Plaque Microbiomes in a Diverse South Florida Population	Study design / Objective	Although it uses supragingival plaque, it is a longitudinal study focused on temporal stability and individuality ("fingerprint"), not primarily designed to compare cross-sectional ethnic structures as the main variable.
Wang et al. 2024	Oral microbiome associated with differential ratios of Porphyromonas gingivalis and Streptococcus cristatus	Sample type / Objective	It focuses on subgingival plaque and the specific proportion of two bacteria (*P. gingivalis* / *S. cristatus*), not on the total community in health.
Chaturvedi et al. 2024	The mouth of America: the oral microbiome profile of the US population	Sample type	It uses oral rinse, not supragingival plaque.
Chaturvedi et al. 2025	Oral Microbiome Profile of the US Population	Sample type	The study was conducted using oral rinse samples, not structured dental biofilm.

Note: This table details the articles excluded during the eligibility phase of the systematic review process (as outlined in the PRISMA flow diagram), specifying the primary methodological or thematic criteria that were not met.

Supplementary Table S 3. GRADE Certainty of Evidence Assessment for the Included Studies.

**Included Study**	**Initial Level of Evidence**	**CRITERIA THAT DECREASE THE SCORE (Downgrade)**					**CRITERIA THAT INCREASE THE SCORE (Upgrade)**		**Final Level of Evidence**
		Study limitations (Risk of Bias)	***Inconsistency***	***Indirectness***	***Imprecision***	***Publication Bias***	***Strength of Association***	***Control of Confounding Factors***	
**Premaraj S, et al., 2020**	Low	Cohorts are homogeneous in terms of number, sex, and origin. No score reduction.	Results are consistent.	No indirectness, as the study is in humans.	Imprecision exists: Wide confidence intervals, and small sample size (<100 subjects) (-2).	Publication bias present.	Associations are strong (+1).	Evaluates confounding factors (+1).	Low quality of evidence
**Zhang Y, et al., 2024**	Low	Evaluated population groups are not homogeneous in lifestyle (-1).	Results are consistent.	No indirectness, as the study is in humans.	No imprecision.	No publication bias.	Associations are strong (+1).	Evaluates confounding factors (+1).	Moderate quality of evidence
**Luo S, et al., 2024**	Low	Sample is homogeneous.	Results are consistent.	No indirectness, as the study is in humans.	No imprecision.	No publication bias.	Associations are strong (+1).	Evaluates confounding factors (+1).	High quality of evidence
**Nath S, et al., 2024**	Low	Sample is homogeneous.	Results are consistent.	No indirectness, as the study is in humans.	No imprecision.	No publication bias.	Associations are strong (+1).	Confounding variables such as age and lifestyle differences were not considered (-1).	High quality of evidence
**Wang Q, et al., 2024**	Low	Sample is homogeneous.	Results are consistent.	No indirectness, as the study is in humans.	Imprecision exists: Wide confidence intervals, and small sample size between groups (-2).	No publication bias.	Associations are strong (+1).	Evaluates confounding factors (+1).	Very low quality of evidence

**Miao X, et al., 2025**	Low	Sample is homogeneous.	Results are consistent.	No indirectness, as the study is in humans.	No imprecision, but the sample size is small (-1).		Associations are strong (+1).	Stratifies by confounding factors (+1).	Moderate quality of evidence

Note: GRADE = Grading of Recommendations Assessment, Development and Evaluation. In accordance with the GRADE framework, observational cross-sectional studies inherently start with an initial "Low" certainty of evidence. The certainty was subsequently downgraded (-1 or -2) due to domains such as imprecision (e.g., small sample sizes, wide confidence intervals) or specific methodological limitations, and upgraded (+1) due to strong magnitude of association and rigorous control of confounding factors. The final level reflects the overall confidence in the taxonomic and functional outcomes reported by each study.

Supplementary Table S4. Methodological characteristics, certainty of evidence (GRADE), and core microbiome profiles of the included studies.

Study (Year) and GRADE Quality	Ethno-racial groups analyzed	Age Range	Sequencing Method	Consolidated Core Microbiome
**Luo S, et al., 2024**	Miao, Han, Zhuang, etc. (China)	9 to 18 years	Full-length 16S rRNA	*Streptococcus, Neisseria, Aggregatibacter, Haemophilus, Capnocytophaga*
**Nath S, et al., 2024**	Australian Natives vs. Immigrants (Australia)	> 18 years (Mean: 31.6 years)	16S amplicon sequencing	*Streptococcus, Corynebacterium, Actinomyces, Leptotrichia, Fusobacterium, Capnocytophaga*
**Zhang Y, et al., 2024**	Zhuang vs. Han (China)	4 to 5 years	16S rDNA (V4 Region)	Proteobacteria, Firmicutes, Bacteroidetes. Genera: *Streptococcus, Neisseria, Haemophilus.*
**Miao X, et al., 2025**	Han (Healthy adolescents with orthodontics, China)	12 years	16S rRNA (V3-V4 Region)	*Leptotrichia, Neisseria, Streptococcus, Fusobacterium, Corynebacterium*

Note: This table synthesizes the dominant taxonomic configurations and technical specifications of the selected primary studies. Bacterial genera are presented in italics according to taxonomic nomenclature standards. GRADE: Grading of Recommendations Assessment, Development and Evaluation; 16S rRNA: 16S ribosomal RNA gene sequencing; Full-length: Full-length sequencing; V3-V4/V4: Hypervariable regions of the 16S gene.
